# Cheek-splitting technique for marginal mandibulectomy: A novel approach

**DOI:** 10.4317/jced.55872

**Published:** 2019-07-01

**Authors:** Atsushi Abe, Kenichi Kurita, Hiroki Hayashi, Yu Ito

**Affiliations:** 1 DDS, PhD. Chief, Department of Oral and Maxillofacial Surgery, Nagoya Ekisai Hospital, Nagoya, Japan; 2 DDS, PhD. Professor, Department of Oral and Maxillofacial Surgery, Aichi-Gakuin University, Nagoya, Japan; 3 DDS. Chief, Department of Oral and Maxillofacial Surgery, Nagoya Ekisai Hospital, Nagoya, Japan

## Abstract

When performing marginal mandibulectomy, ensuring complete tumor removal and preventing postoperative iatrogenic mandibular fracture are essential. Pathological fracture can result due to stress concentration at the site requiring acute angle resection. To perform marginal mandibulectomy without making acute angles in patients with a lesion in the molar or more posterior region, a submandibular or transbuccal approach is necessary. Compared to the submandibular approach, the transbuccal approach is considered useful as it reduces operative time and prevents injury to the facial and mental nerves. Additionally, this approach does not leave a scar in the surgical field, which is beneficial in subsequent neck dissection for late neck metastasis. Here, we report 2 cases of lower gingival carcinoma in which satisfactory aesthetic outcomes were achieved with an improved cheek-splitting technique for marginal mandibulectomy using a transbuccal approach, taking into consideration the angle of the mouth, design of the triangular flap, and modiolus.

** Key words:**Mandibular gingival carcinoma, cheek-splitting technique, transbuccal approach, marginal mandibulectomy.

## Introduction

The surgical method for lower gingival carcinoma is determined according to histological type, cancer progression, and bone invasion and includes marginal mandibulectomy, segmental mandibulectomy, and hemimandibulectomy (1-3). Marginal mandibulectomy approaches to the surgical field include an intraoral approach, submandibular approach, and a transbuccal approach. It is difficult to perform intraoral resection of a tumor located in the posterior region, a submandibular or transbuccal approach is used in such cases. However, there may be room to improve the design of incision lines.Herein, we report 2 cases of lower gingival carcinoma with successful performance of marginal mandibulectomy via a transbuccal approach with an improved incision line for cheek splitting.

## Case Report

-Surgical technique

**Figure 1:**
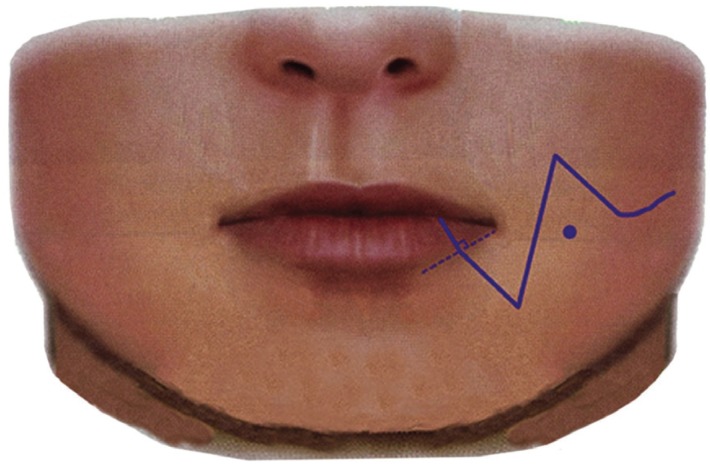
Incision line.Points for designing the incision line include the following: 1) the line should be orthogonal to the vermilion border; 2) the triangular flap must be created so that the bottom length is 1 cm and the apex is at least 5 mm from the mental foramen in a direction toward the midline; 3) the line should pass l cm lateral to the corner of the mouth; and 4) the line should not be made immediately above the modiolus. ● Modiolus.

The incision line has been presented in Fig. 1. The skin incision started 1 cm from the angle of the mouth orthogonal to the vermilion border (the border between the red and white skin of the lip), and, then, a Z-shaped incision was made. The triangular flap was designed so that the bottom length was 1 cm and the apex was at least 5 cm from the mental foramen toward the midline. Subsequently, we created a triangular flap passing l cm lateral to the angle of the mouth, which was designed so that the incision line of the triangle flap was not made immediately above the modiolus. Next, we placed an incision parallel to the mandibular margin up to the resection margin in the oral cavity. When performing an intraoral incision, an incision was made below the parotid duct to avoid injury or extension beyond the anterior edge of the masseter. When performing mandibulectomy, a reciprocating saw was used to secure a sufficiently safe margin.

-Case 1

**Figure 2:**
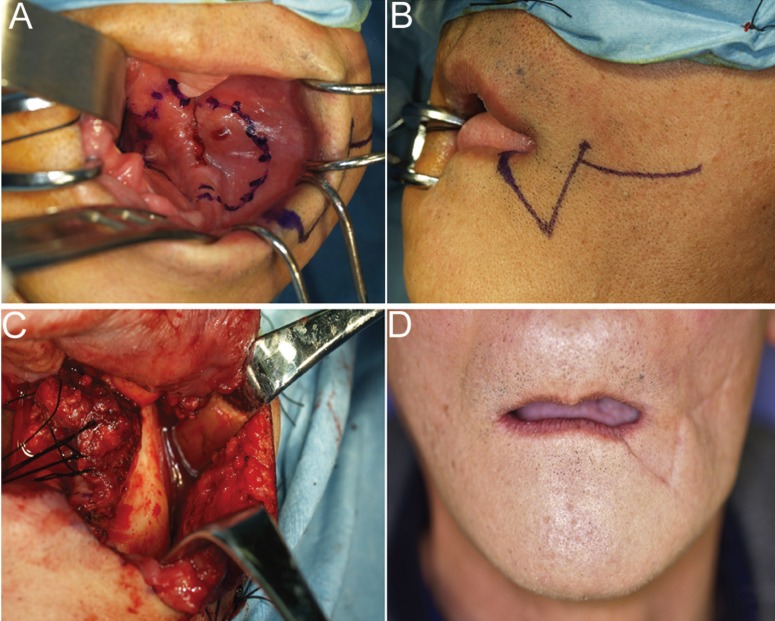
Case 1. A 58-year-old man. A bleeding ulcer 20 × 10 mm in size with induration was observed in the left mandibular molar region. The clinical diagnosis was T2N0M0 (stage II) (Fig. 2A). The incision line for the cheek is shown (Fig. 2B). Marginal mandibulectomy was performed via a transbuccal approach. Tumor resection was performed via a curved incision (Fig. 2C). At 1 year postoperatively, no scar was visible and the appearance was aesthetically acceptable. (Fig. 2D).

A 58-year-old man visited our hospital, where he was completely edentulous on presentation, with a bleeding ulcer 20 × 19 mm in size with surrounding induration in the gingiva of the left mandibular molar region (Fig. 2A), and the submandibular lymph nodes were not palpable. There was no evidence of bone destruction and no submandibular or cervical lymph node or distant metastases by some image inspection. Biopsy was performed, and the lesion was diagnosed as squamous cell carcinoma, with a clinical diagnosis of T2N0M0 (stage II). Marginal mandibulectomy was performed via a transbuccal approach (Fig. 2 B,C). Postoperatively, the patient had no facial nerve palsy, damage to the parotid duct, or inferior alveolar nerve palsy. The patient’s maximal mouth opening was 35 mm, with no difficulty in his daily life (Fig. 2D). The patient had regular postoperative follow-up examinations. Informed consent was obtained from the patient’s parents prior to study initiation, and all procedures were performed in accordance with the Declaration of Helsinki. This report was approved by the Nagoya Ekisai Hospital Ethics Committee (approval number 2018-009).

-Case 2

**Figure 3:**
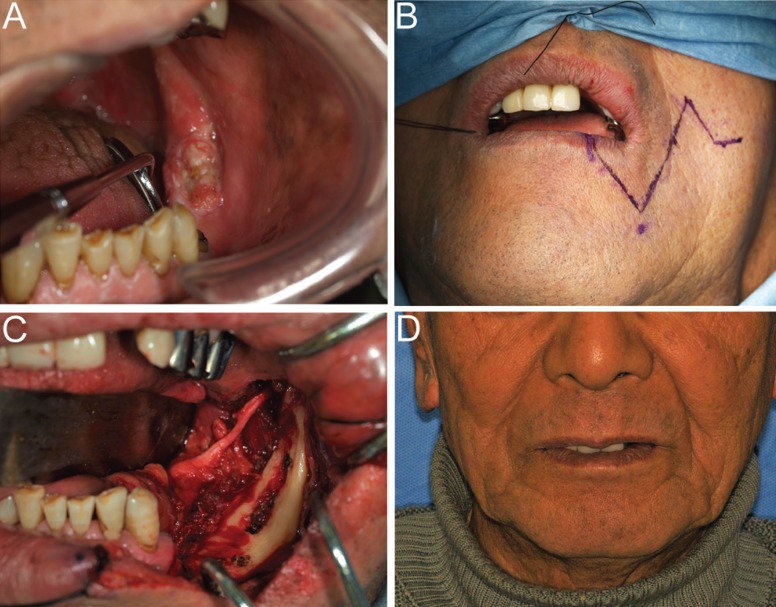
Case 2. An 83-year-old man. An indurated granulated ulcer 10 × 15 mm in size was observed in the left mandibular molar region. The clinical diagnosis was T1N0M0 (stage I) (Fig. 3A). The incision line on the left cheek is shown (Fig. 3B). Tumor resection was performed via a slightly curved incision. (Fig. 3C). At 4 years after surgery, no scar was visible and the appearance was aesthetically acceptable. (Fig. 3D).

An 83-year-old man had noticed a gingival mass in the left mandible since approximately April 2014. The size of the mass subsequently increased, prompting him to visit our hospital. At the first presentation, the patient had an indurated ulcer 10 × 15 mm in size with granular appearance in the left mandibular molar region (Fig. 3A). Neither the submandibular nor the cervical lymph nodes were palpable. Histological diagnosis of the lesion on biopsy revealed squamous cell carcinoma. No evidence of bone destruction was seen on panoramic X-ray, contrast-enhanced CT, MRI, or PET, and there was no infiltration into the adjacent soft tissues, including the tongue, floor of the mouth, or masseter, thus, confirming that the tumor was confined to the gingiva, with a clinical diagnosis of T1N0M0 (stage I). Marginal mandibulectomy was performed via a transbuccal approach (Fig. 3B,C). A linear scar was visible 4 years postoperatively; however, it was aesthetically acceptable, and the clinical course was favorable with no pathological fracture, recurrence, or metastasis (Fig. 3 D). Informed consent was obtained from the patient’s parents prior to study initiation, and all procedures were performed in accordance with the Declaration of Helsinki.

## Discussion

Completely removing the lesions and avoiding iatrogenic mandibular fracture due to stress concentration is important when performing marginal mandibulectomy (1-4). To prevent such fracture, a curved resection is necessary by placing the saw blade perpendicular to the cortical bone of the mandible. Therefore, these cases require either a transbuccal or submandibular approach, allowing the surgical tool direct access to secure the visual field, thereby leading to adequate tumor resection. The advantages of the transbuccal approach are as follows: 1) it can avoid damage to the facial artery and marginal mandibular branch of the facial nerve because there is no surgical invasion to the neck; 2) it allows adequate treatment of the mandibular bone by securing the visual field from the lateral side (5,6).

Conversely, the disadvantages of the transbuccal approach are that care must be taken not to damage the parotid duct or buccal branch of the facial nerve. Additionally, there are concerns regarding postoperative deformity such as incision lines crossing wrinkle lines and difficulty reconstructing the angle of the mouth. We review the key points for improving aesthetic results and preventing postoperative deformity.The first point is the design of the angle of the mouth and lip vermilion. The incision starts 1 cm from the angle of the mouth, preventing surgical wound dehiscence due to the mouth’s opening and closing movement, and alleviating impaired blood flow at the angle of the mouth. The incision line is made at a right angle to the vermilion border line to the vermilion border (the border between the red and white skin of lip) to prevent postoperative gap.

The second point is the design of the triangular flap, essential in avoiding injury to the mental nerve and achieving a good aesthetic appearance. The first triangular flap is designed with the apex at least 5 mm from the mental foramen in a direction toward the midline to avoid injury to the mental nerve and resultant sensory impairment of the lower lip. Additionally, it should be designed so that the incision line is not made immediately above the modiolus. The modiolus is the convergence point of the cheek, orbicularis oris, levator anguli oris, zygomaticus, and depressor anguli oris muscles, and is attached (7). The location of the modiolus is reported to be 11.0 mm ± 2.6 mm (mean ± SD) lateral and 8.9 mm ± 2.8 mm inferior to the cheilion (8,9). Additionally, the facial artery and its branches are present in the modiolus (10). Damage to the modiolus causes loss of denture retention and impaired lip movement and articulation due to tension imbalance between the bilateral modiolus; therefore, it should be preserved without removal.

Using the present method, we were able to obtain a frontal view of the lesion, thus avoiding injury to the lingual nerve with the tip of the reciprocating saw, and perform resection through a slightly curved incision, successfully preventing pathological fracture. The 2 patients presented herein had less facial scarring with sufficient mouth opening and an aesthetically satisfactory appearance.

This approach has not been widely used due to these aesthetic problems; however, it appears to be useful when plastic surgery is required, such as an incision along a skin cleavage line, in carefully selected patients. Marginal mandibulectomy performed via a transbuccal approach can prevent iatrogenic mandibular fracture in cases of posterior oral cavity lesions in patients with a limited surgical field. To minimize postoperative scarring and other aesthetic problems, careful design of the incision line is required, taking into account the vermilion border, angle of the mouth, and modiolus.
